# An intervention to improve antibiotic prescription behavior in veterinary students: A protocol based on the multi‐theory model to tackle antimicrobial resistance

**DOI:** 10.1002/hsr2.1886

**Published:** 2024-02-13

**Authors:** Laleh Hassani, Razie Toghroli, Teamur Aghamolaei, Hamid Sharifi, Maziar Jajarmi, Manoj Sharma

**Affiliations:** ^1^ Social Determinants in Health Promotion Research Center, Hormozgan Health Institute Hormozgan University of Medical Sciences Bandar Abbas Iran; ^2^ HIV/STI Surveillance Research Center, and WHO Collaborating Center for HIV Surveillance, Institute for Futures Studies in Health Kerman University of Medical Sciences Kerman Iran; ^3^ Department of Pathobiology, Faculty of Veterinary Medicine Shahid Bahonar University of Kerman Kerman Iran; ^4^ Department of Internal Medicine, Kirk Kerkorian School of Medicine University of Nevada, Las Vegas (UNLV) Las Vegas NV United States

**Keywords:** antimicrobial resistance, education, interdisciplinary, intervention, mixed‐method, prescription, veterinary medicine

## Abstract

**Background and Aims:**

Antimicrobial resistance (AMR) is a global health threat. Moreover, incorrect and inappropriate drug prescription behavior is considered a fundamental risk factor. Thus, the present study aims to develop, implement, and evaluate the effectiveness of an educational program based on the multi‐theoretical model (MTM) in improving antibiotic prescription behavior in veterinary students of Iran.

**Methods:**

The present study will include four phases including a qualitative phase, an instrument design and psychometric test phase, and a cross‐sectional, and an interventional phase. In the first phase, the sampling will be purposive with a maximum variety. The interviews will be conducted with a sample of veterinarians.

**Results:**

The data will be analyzed in MAXQDA 10. In the second phase, the face and content validity will be tested by a panel of experts as field specialists. A confirmatory factor analysis will be used to test construct validity, and Cronbach's alpha coefficient and intracluster correlation coefficient will be used to determine the internal consistency of the instrument. Then, at this stage, a number of veterinary students will be selected through a multi‐stage sampling method. In the cross‐sectional phase, another sample of veterinary students will complete a researcher‐made questionnaire. Then, Spearman's correlation coefficient test will be used to test the relationship between the two stages of behavior initiation and behavior continuation. The data will be analyzed in SPSS 22. In the third phase, some veterinary students will be selected through a census and will be randomly divided into a control and an intervention group. To collect data in the final phase, the researcher‐made questionnaire that was designed in the second phase of the study based on a multi‐theory model will be used to extract data. To compare demographic characteristics, compare the correlation between the constructs of the multi‐theory model with antibiotic prescribing behavior in the cross‐sectional phase and compare the scores of the constructs of the MTM in two intervention and control groups paired‐samples *T* test and independent‐samples *T* test will be used.

**Conclusion:**

The present study will aim to improve antibiotic prescription behavior in veterinary students based on a MTM. The findings can be used as a model for training students in clinical fields such as veterinary medicine and general medicine at university at a national level. After verification and approval by experts and university professors, we can expect a change in the educational curriculum to include instructions on how to write out prescriptions for students. There are hopes that the present study if conducted accurately and widely to help prevent AMR in livestock, humans, and society.

## 1 BACKGROUND AND AIMS

Antimicrobial resistance (AMR) is a major general health issue arising from an incorrect use of antibiotics in both medical and agricultural sciences. It imposes high economic costs and losses on societies and the healthcare system.^1^ As reported by the World Health Organization (WHO), AMR occurs when microorganisms (e.g., bacteria, fungi, viruses, and parasites) are exposed to antimicrobial drugs (e.g., antibacterials, antifungals, antivirals, antimicrobials, and so on). Microorganisms that develop AMR counteract the effect of drugs on the body; thus, infections remain in the body and increase the risk of spreading infections to others. More than 700,000 mortalities are reported annually due to AMR. As predicted by 2050, if no measure is taken, AMR will turn into a major cause of mortality on a global scale. The mortality rate caused by microbial resistance is higher than the total number of deaths induced by cancer worldwide. Yet, the former has been largely neglected and, instead, issues such as cancer and how to treat it have been addressed more.^2^, ^3^

Despite the beneficial effects of antibiotics on the treatment of livestock infectious diseases, the presence of their residues in milk and animal meat, as well as their transfer to the human body have adverse effects on health, industry, and economics. As reported by the National Center for Rational Prescription of Antibiotics, consuming antibiotics in Iran is 16 times as high as the global standard. Some researchers believe that the spread of microbial resistance to antibiotics results not only from the unnecessary prescription and use of these compounds in humans but also from the widespread use of antimicrobial drugs in veterinary medicine. It has caused the transfer of such pathogenic bacteria from animals to human pathogens. The main difference between microbial resistance to antimicrobial drugs in humans and animals is that microbial resistance in humans affects the individual, whereas microbial resistance in livestock affects a large population due to the consumption of raw animal products by humans. Exposure to both resistant bacteria and antibiotic compounds prescribed for the treatment of infectious diseases for livestock through transmission causes the accumulation of drugs and drug residues in raw livestock products.^4^

**Figure 1:**
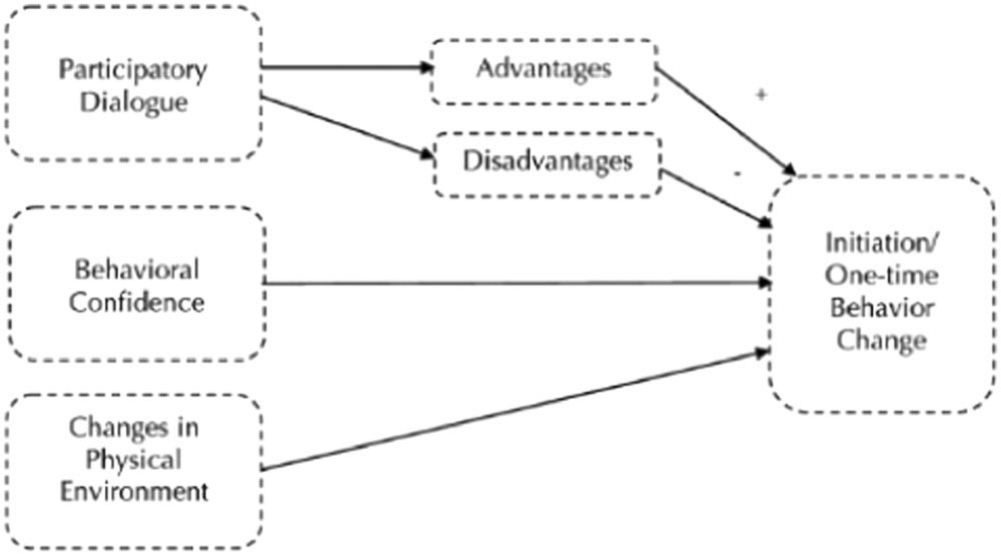
Constructs in the initiation of health behavior change in the multi‐theory model (MTM) of health behavior change.

**Figure 2:**
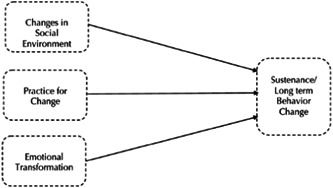
Constructs in the sustenance of health behavior change in the multi‐theory model (MTM) of health behavior change.

It seems that attempts to prevent the occurrence of microbial resistance in livestock and its consequences for humans are effective and can be implemented efficiently by veterinarians and those active in this domain. What veterinarians can do with this respect is wide‐ranging. Yet, it is hard to make interventions with veterinarians directly involved because they are not easily available for research; therefore, veterinary students are the best and closest population for interventional studies. If this population adequately understand the principles of prescribing antibiotics, this successful learning will be productive in practice too.^5^ The multi‐theoretical model (MTM) is a health behavior theory that uses a fourth‐generation framework to predict the initiation and sustenance of healthy behaviors.^6^ In the initiation model (short‐term changes), three main constructs describe the changes in health‐related behaviors, which include participatory dialog, behavioral confidence, and changes in the physical environment.^7^, ^8^ In addition, MTM includes three other constructs that influence the sustenance of a healthy behavior change. These constructs include emotional transformation, practice for change, and change in the social environment.^9^, ^10^ Constructs in the initiation and sustenance of health behavior change in the multi‐theory model (MTM) of health behavior change are presented in Figures 1 and 2. The use of MTM has consistently shown its highly predictive power in several health‐related behaviors.^9^ Motivated by the growing importance of AMR in the world and Iran, and the importance of training and improving veterinarians’ behavior as an influential population in preventing the issue, the present study was conducted. Due to a lack of interventional research on this topic, the present research aimed to develop an intervention to improve antibiotic prescription behavior in veterinary students of the Bahonar University of Kerman with an integrated health approach and in an interdisciplinary and multistage manner. Many studies have mentioned the importance of AMR and its impact on global health.^11^ However, most of the studies in this field are limited to laboratory studies, and the lack of community‐based studies and interventions regarding antibiotic prescription behavior in veterinarians and other health workers to prevent AMR is clearly felt.^12^ Therefore, considering the importance of the AMR issue and the lack of intervention research by the One Health Approach, the need for innovative interdisciplinary studies in this area is necessary.^13^, ^14^

### 1.1 Objectives and study

1. To identify the participants’ experiences and views of antibiotic prescription and the barriers they face.
2. To design an appropriate instrument with items derived from in‐depth semistructured interviews in the qualitative phase.
3. To implement the cross‐sectional phase to verify the instrument to measure antibiotic prescription behavior and to check the predictability of the model constructs.
4. To implement the intervention following the effect of the determinants of the model constructs.

## 2 MATERIAL AND METHOD

The present mixed research will be done in four phases: qualitative, instrument design and psychometric testing, cross‐sectional, and interventional phases.

To design the present study, a team of experts was formed including health education experts, veterinarians active in clinical work, veterinary professors, and statisticians.

In the first phase of the study, a number of veterinarians will be included in the qualitative phase. They will be asked about their experiences and opinions of antibiotic prescription and the perceived barriers to preventing AMR and achieving the appropriate antibiotic prescription. Then, the information from the first phase will be used to develop a researcher‐made questionnaire. The second phase also involves testing the psychometric qualities of the instrument, its validity, and reliability. In the third phase (cross‐sectional phase), the constructs that determine behavior change and the interrelationship of the constructs will be tested. In the fourth phase (intervention), based on the information gained from the cross‐sectional phase, an educational intervention will be developed.

The approximate timeline of the project is presented in Table 1.

**Table 1:** Project's timeline.

Phase	Activities	Duration (month)
Qualitative phase	Semistructured face‐to‐face interviews with veterinarians	3 months
Instrument design and psychometrics	− Developing an initial draft of the questionnaire based on the data from face‐to‐face interviews in the previous phase − Removing duplicates, merging, and modifying items − Testing the content validity and face validity of the initially proposed questionnaire − Testing the construct validity (through exploratory and confirmatory factor analysis) − Testing the reliability	3–4 months
Cross‐sectional phase	− Distributing the researcher‐made questionnaire among the student and intern populations − Determining the most effective constructs to develop training sessions for the intervention phase − Testing the strength of correlation between constructs	4 months
Intervention phase	− A pretest survey − Educational needs assessment based on the information obtained from the pretest − Developing an educational intervention (determining the number of sessions, the duration of each session, resources, human resources, budget, educational materials, and so on) − Implementing the program—holding intervention sessions based on the intervention plan designed in the previous phase	7 months
Waiting time to evaluate behavioral changes	− Evaluating the results (in the posttest survey 3 months after the intervention)	3 months

### 2.1 Participants

The research population is Iranian veterinarians in the qualitative phase and Iranian students of veterinary internships in the cross‐sectional and interventional phases. To select the participants in different steps of the study, we will use some inclusion and exclusion criteria. These criteria are presented in Table 2.

**Table 2:** Inclusion and exclusion criteria.

Phase	Inclusion criteria	Exclusion criteria
Phase 1	(1) Being a clinically active veterinarian (in the public and private sectors) (2) Having an experience of at least 1 year in veterinary medicine (3) Participating in the interview and sharing experiences (4) Having rich and useful experiences with drugs, prescribing antibiotics and treating infectious veterinary diseases, and willingness to share experiences with the researcher	(1) Unwillingness to continue the interview (2) Not signing the written consent
Phase 3, 4, 5	(1) Willingness to participate and sign an informed written consent (2) Being a student of the veterinary medicine internship (3) Access to WhatsApp or Telegram virtual networks	(1) Incomplete questionnaires (2) Not participating in training sessions for more than one session (intervention phase) (3) Not signing the informed letter of consent

### 2.2 Phase 1: Qualitative phase

#### 2.2.1 Design of study

The purpose of this phase is to explore the participants’ experiences and views of antibiotic prescription, and the barriers they face to the prevention of AMR and appropriate antibiotic prescription. In the qualitative phase, we will invite the participants to participate in the interview by email and we will continue sampling until data saturation. Data saturation is the point in the research process where enough data have been collected to draw the necessary conclusions and any further data collection will not produce value‐added insights. In the following, the data obtained from this phase will be used to design a research questionnaire based on the multi‐theory model.

For data analysis, directed or theory‐based qualitative content analysis will be used, including:

− transcribing the interviews
− reading the transcripts verbatim
− extracting the main themes
− assigning abbreviated codes
− assigning codes to the subscales/subcategories
− assigning subscales to the main categories^15^

#### 2.2.2 Sample selection

In the qualitative phase, the participants will be veterinarians from three metropolises of Iran, that is, Bandar Abbas (a fishing and aquaculture hub), Tehran (with a large population of pets and industrial poultry), and Kerman (with a large population of big and small livestock). All participants should have the experience of clinical work and antibiotic prescription. The sampling will be done purposefully with a maximum variety of fieldwork, city of work, age, and so on. In purposive sampling, the participants are selected as a sample to provide the information needed to answer the research questions. The sampling continues until data saturation, that is, until no new information is added.^16^ In the present study, the veterinarians active in the veterinary system will be contacted via email to participate in the study.

Before the interview, a proper relationship will be established with the interviewers (an introduction to the researcher and his educational level, unraveling the purpose of study, assuring the interviewee of the confidentiality of information and recording conversations, bringing reason for selecting the interviewee, obtaining informed consent to record the audio, and so on). This will gain the participants’ trust and further interest them to participate in the study. Having consented to take part in the study and complete the demographic information questionnaire, for audio‐recording conversations, the participant's right to withdraw from the study will be also warranted. The participants also will be assured that the interview content will be completely confidential.

#### 2.2.3 Data collection

The data collection instrument will be a demographic questionnaire and an interview guide. In this phase of the study, in‐depth individual semistructured interviews will be conducted.^17^ Health education and health promotion will be addressed.

The questions will be of three kinds. General questions will be asked such as “When do you prescribe antibiotics?.” More specific questions will be also asked based on the veterinary students’ previous answers. The third kind of questions will be led by the model constructs, such as:

1. What do you think about the benefits and barriers of prescribing antibiotics? (the participatory dialog construct)
2. Do you increase your ability to control the unnecessary prescription of antibiotics? (the behavioral confidence construct)
3. How do you aim to control antibiotic prescription behavior in yourself? (the perceived behavioral control construct)
4. Is it possible to provide a way not to get antibiotics other than veterinary pharmacies? (the change in physical environment construct)
5. Can you control the rapid prescription of antibiotics in yourself? (the emotional transformation construct)
6. Is there no other way than to quickly prescribe antibiotics? If yes, why is it not applied? what is the problem? (the practice for change construct)
7. Can you think of a way to avoid easy access to antibiotics? (The change in the social environment construct) and other questions if needed:
8. What measures do you take to control the degree of antibiotic prescription in yourself?
9. How is drug administration affected by different factors?
10. What are the most important factors influencing your prescription of antibiotics?
11. Can you name some factors that have contributed to the prescription of antibiotics?
12. What do you currently do to improve your medication prescription behavior?
13. What do you think are the facilitators of appropriate drug administration?
14. What factors can influence your quality of prescription?
15. What was your experience of dealing with an antibiotic disease or a patient resistant to antibiotic treatment?
16. What are the problems facing veterinarians in society at the individual, organizational and policy‐making levels for prescribing medicine?

To have a better exchange of ideas and information, the interviews will be held face‐to‐face. The interview will be conducted in a short period of time in order for the interviewees to feel comfortable and less mentally obsessed. During the interview, the desired parts will be noted and audio‐recorded. Transcripts will be made shortly after the interview. The next questions will be exploratory and the participants (to clarify their experiences and opinions) will be asked to share their experiences with examples.

#### 2.2.4 Rigor

To ensure the reliability of findings, the researcher will use Lincoln and Guba's criteria^18^:

(1) Manuscripts and the extracted codes will be reviewed by the participants. (2) Data will be sent to qualitative researchers and revised based on additional comments. (3) Sufficient time will be devoted and a continuous review will follow. (4) Participants will be selected with a maximum variety. (5) The participants’ research process and characteristics will be described and examined in detail.

#### 2.2.5 Data analysis

After each interview, the transcribed information will be analyzed simultaneously, taking into account nonverbal communication and categorization. Data analysis (coding and continuous comparison) and note‐taking will be next. This step can continue until data saturation. When the data are saturated, the sampling is over; otherwise, it will go back to the participant selection phase and these steps will be repeated.

The data analysis will begin by listening to the participants’ descriptions and will continue by recurrently rereading the data until the main themes are derived. Exploring the underlying relationships among statements and providing a comprehensive description of the phenomenon will be the final step. The transcripts will enter MAXQDA‐10 to manage and code the data.

### 2.3 Phase 2: Instrument design and psychometric test

The purpose of this phase of the study is to test the psychometrics and validity of the questionnaire designed based on the data obtained from the previous phase. After extracting themes, codes, and main categories, the constituent items of the questionnaire will be constructed. Duplicate items should be omitted and similar items can be merged. Then the validity and reliability of the instrument will be measured. To test the former, the three methods of face validity, content validity, and construct validity will follow. To test the latter, Cronbach's *α* test of internal consistency^19^ will be used as well as the test‐retest method.

#### 2.3.1 Sample selection

Having analyzed the comments and designed the initial draft of the instrument, the questionnaire will be provided to some veterinary medicine trainees and interns (for each item of the qualitative phase questionnaire, five people will be selected for this phase). The sample size is estimated with an error of 5%, a power of 80%, and an effect size of 08/0, in G*Power.^20^ To substantiate face validity qualitatively, face‐to‐face interviews will be conducted with a number of participants and the following items will be examined:

− the level of difficulty: identifying items, phrases, or words that are hard to comprehend.
− the level of appropriateness: accuracy, appropriateness, and the desired contribution of items to the main purpose of the subscales of the questionnaire.
− ambiguity: examining the probable wrong understandings of phrases, items, or inadequacy of the meanings of words.^21^, ^22^

After correcting the items, based on the participants’ comments in this phase of the study, in the next step, the item impact quantitative method will be used to remove inappropriate items and determine the relative importance of each item. Many researchers recommend at least two or three experts for questionnaire review, so in this phase, a panel of 10 experts from different specialties of veterinary medicine, health education, health promotion, and qualitative research experts will be consulted for psychometrics and validation of the instrument.^23^ To quantitatively test face validity, the sample size required for confirmatory factor analysis was determined for each item.^24^ Confirmatory factor analysis will be used to check the validity of the model constructs.

#### 2.3.2 Data collection

Online questionnaires will be used for data collection.

The questionnaire will be designed in cyberspace and the hyperlink will be shared with students via WhatsApp.

Then, it will be completed by veterinary students as self‐reports, in one session after fully informed consent to do so. The Porsline online platform will be used for data collection.

#### 2.3.3 Data analysis

The data will be analyzed in SPSS‐22 and EQS. 6.1. To test the validity of model constructs, confirmatory factor analysis will be used in EQS. 6.1.

### 2.4 Phase 3: Cross‐sectional phase

The cross‐sectional phase aims to explore the constructs with the greatest impact on the intervention and also test the strength of association between the constructs. The sample size is estimated with an error of 5%, a power of 80%, and an effect size of 08/0, in G*Power.

#### 2.4.1 Sample selection

For the cross‐sectional phase, the sample will be selected from the veterinary internship students who have begun prescribing medicine at the universities of Tehran, Shiraz, and Zabul.

#### 2.4.2 Data collection

The information needed to do the cross‐sectional phase will be collected using a researcher‐made questionnaire in two parts:

1. Demographic information (the student's age, academic year, semester, weekly study hours, and so on).
2. A researcher‐made questionnaire to measure the correct antibiotic prescription behavior based on the MTM constructs.

The scoring of the questions will be done on a 5‐point Likert scale (*strongly agree* = 5, *agree* = 4, *undecided* = 3, *disagree* = 2, *strongly disagree* = 1). A higher score will indicate a favorable state of a given construct.^25^

The subscales include questions related to participatory dialog, behavioral confidence, change in the physical environment, emotional transformation, practice for change, and change in the social environment.

#### 2.4.3 Data analysis

Quantitative data description will use frequency tables, graphs, and central and distribution indicators. Qualitative data description will use frequency tables. To analyze the data, test the relationships, and make comparisons, multivariate analysis of variance, multivariate multiple linear regression, and Pearson's correlation test will be used. All analyses will be done in SPSS 22, and in all tests, a significance level of 0.05 will be considered. Amos will be used to identify the predictive power of each construct in the target behavior, the theoretical model, and the relationships of these variables with each other and with the target behaviors.

### 2.5 Phase 4: Interventional phase

#### 2.5.1 Design of study

The present quasi‐experimental interventional study will be a clinical trial registered as IRCT20210911052432N1. The aim will be to test the effect of the MTM of health behavior change intervention on the improvement of antibiotic prescription behavior of veterinary doctoral intern students of Bahonar University in Kerman and Ferdowsi University of Mashhad.

#### 2.5.2 Sample selection

In this phase of the study, the sample needed for the intervention group will be selected from among the veterinary students of these two universities in Iran, who are engaged in internships in the academic year 2022–2023, meeting the inclusion criteria as a census. Then, through a random allocation, they will be assigned to the intervention and control groups. The intervention group and the control participants will selected by a census.

#### 2.5.3 Data collection

The instruments used in this phase will be a demographic questionnaire and the final version of a questionnaire related to prescription behaviors, whose validity and reliability be measured in the second phase. In the intervention phase, due to the small number of students in the field of veterinary medicine in Iran's colleges and to avoid bias error and peer influence, the students will be enrolled in the study by census method. As full follow‐up of students is required in the intervention phase, the intervention will start at the beginning of the academic semester and will end shortly before the end of it. As a result of following up on how to answer questionnaires in both control and intervention groups, the amount and quality of students’ participation in training sessions in the intervention group will be carefully and completely monitored and followed up.

#### 2.5.4 Data analysis

Data analysis will be done in SPSS 22. The distribution of demographic variables in intervention and control groups will be compared using the independent‐sample *T* test and *χ* ^2^ test. To compare the mean scores of prescriptive behaviors and the MTM constructs (participatory dialog, behavioral confidence, changes in the physical environment, emotional transformation, practice for change, change in the social environment) before the intervention and after the intervention the Pairwise or Wilcoxon's nonparametric test will be used. Independent‐sample t‐test or its equivalent nonparametric Mann–Whitney *U* test will be used to compare the scores of constructs and behavior between the two groups. Also, covariance analysis and multivariate analysis will be used for data analysis. All findings are interpreted at a significance level of *p* < 0.05.

#### 2.5.5 Intervention design

Students and interns in the intervention group will participate in a diagnostic evaluation test based on the educational needs assessed through: (a) watching an educational video, (b) reading pamphlets and educational booklets about AMR and improved prescription behaviors in students, (c) participation in all mandatory training sessions for participants, (d) 15 theoretical training sessions and three practical training sessions in a laboratory to teach diagnostic techniques, and (e) a phone‐mediated follow‐up. Different methods will be used to teach the students of the intervention group. These will be student‐centered communication, unambiguous communication techniques, practical methods of laboratory diagnosis for students, and so on, which will probably lead to better results in the intervention group. Participants in both groups will be evaluated 3 months after the intervention.

### 2.6 Intervention strategies

#### 2.6.1 Participatory dialog

This construct is based on two‐way communication with an emphasis on the advantages and disadvantages of changing health‐related behavior.^6^ It considers the pros and cons of changing the behavior and emphasizes collaborative communication that can be practiced by professors of the University of Basic and Clinical Sciences and Health educators.

During the training program, learners will be helped to express their feelings and moods about the prescribed behaviors to better understand and be able to make the necessary changes to improve their health.

#### 2.6.2 Behavioral confidence

This construct may come from external sources such as specific individuals or populations during the academic years, whereas at work, or in life, such as health educators, professors, researchers, scientists, and so on. This construct attempts to measure the extent to which someone will engage in health‐related behavior change in the future rather than now. The issue lies in one's confidence in the ability to show the appropriate behavior of diagnosing and prescribing drugs through multiple steps, including an initial clinical diagnosis, sending the sample to a laboratory, relying on the laboratory diagnosis, and requesting a test. Antibiogram, laboratory results drug prescription, and scientific and practical familiarization with new and popular methods on a global scale for drug prescription will be needed. Confidence‐building behaviors and implementation by students will be recorded to provide feedback.

#### 2.6.3 Changes in the physical environment

This construct is specific to the physical environment and not the social one. Changes in the physical environment include interference and modifications in resource availability, accessibility, and sustainability. These changes are possible through facilitating students’ access to laboratory resources, clinical and clinical diagnosis, and therapeutic methods, new laboratory tests including bacteriology and virology, providing resources, and allowing all students and veterinarians to access different parts of a laboratory, resources, laboratory materials, and devices.

#### 2.6.4 Emotional transformation

This construct involves changing emotions and directing them to help change health behaviors. This construct emphasizes active reflection and reflective behavior. Emotional transformation can be achieved through dialog, brainstorming, experimental projects, and group organization. These dialogs can be advanced through discussions in multiperson groups and online discussions. Awareness can be raised through projecting and role playing, simulation, and team work. Also, to practice change to achieve emotional transformations, opportunities for developing practical plans or implementing laboratory projects can be facilitated. The transformation construct can be studied through case studies, field meetings, and the like. Also, the formation of groups, coalitions, and the formation of organizations can help.

#### 2.6.5 Practice for change

This construct involves incorporating ongoing changes to eliminate ineffective strategies, remove barriers, focus on health behavior change, address barriers, and focus more on health behavior change. The training will be done with an emphasis on highlighting the threats of the disease or the severity of conditions for students. The negative consequences of the disease and the high costs of hospitalization and treatment, the impact of the disease on the socioeconomic status and quality of life will be addressed. The content will be presented to reduce the costs of the new behavior, which will include the benefits of using new methods of antibiotic diagnosis and the advantages of improving prescription behaviors and preventing AMR and its consequences. A pamphlet will be distributed including recommendations to reduce the complications of the disease. Also, brochures and posters illustrating the step‐by‐step diagnosis and treatment of bacterial and viral diseases will be provided for students. Similarly, posters related to laboratory techniques, working with devices and materials, and protocols will be put up for students to see closely in the laboratory. Special prescription sheets will be used with minimal space for one drug (one‐line prescriptions) for the practice of change.

#### 2.6.6 Changes in the social environment

This construct consists of social support, helping relationships, and the environment's constituent elements. Professors, clinical staff and laboratory personnel, university administrative staff, system aid institutions, and veterinary organizations may facilitate changes in the social environment.

## 3 DISCUSSION

Due to the increase in AMR, it seems necessary to develop and implement preventive programs in the target population in Iran. The WHO introduced education as one of the most essential modes of prevention programs. Education will introduce a lasting change to the attitude and performance and help eventually make changes in the lifestyle. Research shows that the most effective educational and analytical programs are based on a theory‐based approach stemming from behavior change methods.^26^ In Iran, little is known about the educational interventions of AMR. The role it can play in improving diseases, in decision‐making, and functioning in the health system is still unclear on a national scale. Considering the significant effect of AMR on society's health, it is essential to pay attention to society's health through an integrated health approach. It is necessary to conduct similar studies to promote a correct understanding of the issue and guide planners, officials, and trustees of the health sector, to provide better systematic services for society.

Considering the importance of AMR, the effect of correct prescription, and veterinarians’ and doctors’ role in preventing and controlling AMR and its effect on patients’ quality of life and treatment of infectious diseases, similar studies will be highly recommended. This issue has been scarcely addressed in Iran and there is no empirical evidence available. The previous theoretical models that have been used to explain the behavior lack an expected predictive power and have failed to account for a long‐term behavior change. Moreover, the MTM model is a very new and comprehensive theory that has shown promising evidence in predicting different types of behaviors. Thus, there are hopes that the current study based on this model and with an interdisciplinary scientific approach can solve the problem of AMR. In a cross‐sectional study, Zareshahi et al.^27^ explored the indicators of rational drug prescription in doctors’ prescriptions in Kerman. The results showed that the existing training processes for doctors’ prescribing indicators are far from global standards and it is recommended to increase the quality and quantity of training programs and implement efficient management and continuous monitoring of the drug prescription behavior and the ability to monitor and control rational drug prescription. Therefore, in the present study, the MTM model will be used to predict the change in students’ behavior to improve the behavior of prescribing antibiotics, so that they develop a sound understanding of the basic behavioral, environmental, social and interpersonal factors involved in behavior change.

## 4 LIMITATIONS AND STRENGTHS OF THE STUDY

Although the random assignment of students to two intervention and control groups leads to the same distribution of confounding variables and prevents selection error, there is a possibility that some students will have more willingness and participation in the current research with more study. Therefore, it will not be possible to generalize this study to all students.

Due to data collection by online questionnaire, it is not possible to explain the questions to the participants. Therefore, at the beginning of the questionnaire, the main researcher will provide his contact number for any possible questions, and in addition, in the explanation section of the questionnaire, he will try to give a brief explanation for some questions. For the participation of the students and their cooperation to adhere to the study, a small booklet will be given to all of them as a gift. Future investigations can include multilevel interventions to provide solutions to combat global threats.

## AUTHOR CONTRIBUTIONS

**Razie Toghroli**: Writing—original draft, investigation, validation, software, supervision, data curation, writing—review and editing, methodology. **Teamur Aghamolaei and Laleh Hassani**: Conceptualization, writing—review and editing, supervision. **Hamid Sharifi**: Visualization, software, formal analysis. **Maziar Jajarmi**: Project administration, resources. **Manoj Sharma**: Supervision.

## CONFLICT OF INTEREST STATEMENT

The authors declare no conflict of interest.

## ETHICS STATEMENT

Ethical approval was received for this study from the Ethics Committee of the Hormozgan University of Medical Sciences (IR.HUMS.REC.1400.207). Written informed consent will be obtained from individuals who participated in this study. The authors confirm that all methods will be carried out in accordance with relevant guidelines and regulations. All experimental protocols were approved by the Ethics Committee of the Hormozgan University of Medical Sciences.

## TRANSPARENCY STATEMENT

The lead author R. Toghroli affirms that this manuscript is an honest, accurate, and transparent account of the study being reported; that no important aspects of the study have been omitted; and that any discrepancies from the study as planned (and, if relevant, registered) have been explained.

## Data Availability

The data that support the findings of this study are available from the corresponding author upon reasonable request.
